# Decrease in incidence of distal radius fractures in Oslo, Norway

**DOI:** 10.1007/s11657-024-01383-6

**Published:** 2024-04-11

**Authors:** I. Oftebro, S. A. Skjaker, H. L. Fridheim, F. Frihagen, H. E. Meyer, L. Nordsletten, L. B. Solberg

**Affiliations:** 1 Department of Orthopedic Surgery, Oslo University Hospital, Nydalen, 0424, Postbox 4950, Oslo, Norway; 2 Institute of Clinical Medicine, University of Oslo, Oslo, Norway; 3 Department of Orthopedic Surgery, Diakonhjemmet, Oslo, Norway; 4 Department of Orthopedic Surgery, Østfold Hospital Trust, Grålum, Norway; 5 Department of Physical Health and Ageing, Norwegian Institute of Public Health, Oslo, Norway; 6 Department of Community Medicine and Global Health, University of Oslo, Oslo, Norway

**Keywords:** Epidemiology, Distal radius fracture, Fragility fracture, Incidence, Oslo

## Abstract

***Summary*:**

This study reported the incidence of validated adult distal radius fractures in Oslo, Norway, in 2019. The incidence has been reduced over the last 20 years. However, it is still high compared to other regions in Norway and some of the other Nordic countries.

**Purpose:**

We aimed to report the incidence of distal radius fractures in Oslo in 2019 and compare it to the incidence rates in 1998/1999.

**Methods:**

Patients aged ≥ 20 years resident in Oslo sustaining a distal radius fracture in 2019 were identified by electronic diagnosis registers, patient protocols, and/or radiology registers. The diagnosis was verified using medical records and/or radiology descriptions. We used the same method as the previous study from Oslo, making the comparison over time more accurate. The age-adjusted incidence rates and the age-standardized incidence rate ratio (IRR) were calculated.

**Results:**

The absolute number of fractures decreased from 1490 in 1998/1999 to 1395 in 2019. The IRR for women and men in the age group ≥ 20 years in 2019 compared to 1998/1999 was 0.77 (95% CI 0.71–0.84) and 0.77 (95% CI 0.66–0.90), respectively. The IRR for women and men in the age group ≥ 50 years in 2019 compared to 1998/1999 was 0.78 (95% CI 0.71–0.86) and 0.78 (95% CI 0.63–0.97), respectively. For the population in Oslo with Asian background compared to Norwegian background in the age group ≥ 50 years, the IRR in 2019 was 0.57 (95% CI 0.40–0.80) for women and 0.77 (95% CI 0.44–1.37) for men.

**Conclusions:**

The incidence of distal radius fractures in Oslo has decreased over the last 20 years. It is still, however, higher than in other areas of Norway and in some of the other Nordic countries.

## Introduction

Fracture of the distal radius is the most frequent fracture in adults [1]. It is often the first fracture indicating osteoporosis in the middle aged and elderly and may be a warning of more fractures to come [2–5]. Distal radius fracture is an increasing economic burden to society due to an ageing population and more complex treatment [6–9]. For patients ≥ 50 years, the distal radius fracture may serve as a sentinel fracture for secondary fracture prevention. Fracture epidemiology may aid in allocating health recourses.

The incidence of distal radius fractures in Oslo, Norway, was studied in 1979 and in 1998/1999. There was no significant change in the incidence of adult distal radius fractures between 1979 and 1998/1999. However, the overall incidence of distal radius fractures in Oslo has been higher than in other European countries [10, 11]. There is little research on the incidence of distal radius fractures in developing countries, and the global variation in the incidence is difficult to interpret given methodological variations in sampling frames and fracture identification [12]. However, from Nordic countries that are similar to Norway, like Sweden, Finland, and Denmark, recent studies have demonstrated lower incidence than the previous studies from Oslo [13–15]. Similar results were found in a study from the neighboring county of Oslo, in 2011 [16].

The aim of this study was to investigate if there has been a change in the age-standardized incidence rate ratios (IRR) of distal radius fractures over the last two decades in women and men age ≥ 20 years in Oslo. We also studied the IRR in those ≥ 50 years and the IRR in one of the largest groups of immigrants in Oslo, people with Asian origin.

## Materials and methods

### Study population

We included patients sustaining a distal radius fracture between January 1 and December 31, 2019, aged ≥ 20 years and Oslo residents according to the National Population Register. Data on the “standard population” as well as immigration status were retrieved from Statistics Norway [17].

### Variables

Distal radius fractures with or without a fracture of the distal part of ulna were included: the International Classification of Diseases 10th Revision (ICD-10)-codes S52.5, S52.6. Date of birth, gender, the patient’s geographical region of origin, date of first visit to a clinic with the fracture, and fracture side were recorded. A bilateral fracture was counted as two fractures. If a patient had two different distal radius fractures within the observation period, it was recorded as two fractures. If patients were seeking medical attention in an outpatient clinic and then referred to a hospital department for the same fracture, they were only recorded once. The patients’ geographical country of origin was defined as the person’s country of birth or the country of birth of the person’s parents or grandparents. Asian origin included all countries in Asia and Turkey.

### Data collection and validation

The patient data were collected from the outpatient clinics and hospitals in Oslo treating distal radius fractures; Oslo University Hospital, Akershus University Hospital, Diakonhjemmet Hospital, and Volvat Medical Centre (the three hospitals are public, while Volvat is a private clinic). Patient administrative systems with electronic diagnosis registers at each hospital/clinic were used to identify all patients with the ICD-10 codes S52.X or S62.8 in the study period. At Volvat Medical Centre, the ICD-10 code M79.6 and the International Classification of Primary Care, 2nd edition (ICPC-2) – codes L74, L76, and L11 were used to identify the patients in addition to S52.X and S62.8 as Volvat used both systems. To verify the diagnosis of distal radius fracture, the clinical charts and radiology descriptions from all identified patients were retrieved. In ambiguous cases, the radiographs were examined by the first author. At the largest site, Oslo University Hospital’s, which treats the vast majority of the patients [11], lists of all patients with a radiograph or CT scan of the wrist or forearm in the period were used to identify additional patients not identified in the electronic diagnosis registers.

### Ethics

The study was approved by Regional Committee for Medical and Health Research Ethics (REK 2018/2559) and by the Data Protection Officer at Oslo University Hospital (19/03873). A request to waive informed consent was approved.

### Statistical analyses

Categorical data were analyzed using Fisher’s exact test and by the Pearson chi-square test. A *p* value < 0.05 was considered statistically significant. The age- and sex-specific incidence rates for 2019 were calculated and compared to 1998/1999 and 1979. The age-adjusted incidence rates in 2019 and 1998/1999 were calculated using a direct standardized method, and the 2019 Oslo population [17] was used as the standard population. Both the whole-year incidence and the summer incidence were reported to compare with the numbers from 1998/1999. The summer incidence was calculated using the fractures occurring in the 6 months from April to September and multiplied by two, giving a hypothetical number of fractures for a year without winter.

Within-stratum Mantel–Haenszel weights were used to find the age-standardized IRR with 95% confidence interval (CI) to compare the incidence rates between the different periods and different country of origin.

STATA SE (Version 17, Stata Inc. North Station, TX, USA) was used for all of the data analyses.

## Results

In total, we identified 1395 distal radius fractures in patients ≥ 20 years of age in 2019 of which 995 (71%) fractures occurred in patients ≥ 50 years. There were 1372 patients as 11 patients had two different episodes with a distal radius fracture and 12 patients sustained bilateral fractures during the study period. There were 75% (*n* = 1048) women, and the median age was 62 for women (range 20–98) and 51 for men (range 20–95) (*p* < 0.001). There were 759 (54%) left-sided fractures, 612 (44%) right-sided fractures, and 12 were bilateral fractures. Ninety percent (*n* = 1247) of the patients were treated at the outpatient clinic at Oslo University Hospital, and 1136 (91%) were found to have a correct diagnosis code, while 111 (9%) were captured either by a control code or by the examination of radiology descriptions.

### Total incidence and incidence in patients ≥ 50 years

The age-adjusted incidence rates per 10,000 observation years for women ≥ 20 years were 48.3 (95% CI 45.4–51.2) in 1998/1999 compared to 37.6 (95% CI 35.3–39.9) in 2019 with the corresponding figures in men being 17.0 (95% CI 15.1–18.8) in 1998/1999 and 13.3 (95% CI 11.9–14.7) in 2019 (Table 1). The age-standardized IRR for women and men in the age group ≥ 20 years in 2019 was 0.77 (95% CI 0.71–0.84) and 0.77 (95% CI 0.66–0.90) compared to 1998/1999. The total number of fractures was 1490 in 1998/1999 and 1395 in 2019.

**Table 1:** The incidence rate of distal radius fractures in different age groups in women and men in 2019

Sex	Age group	Population 01 Jan 19	Number of fractures	Annual incidence per 10,000
Women	20- 30- 40- 50- 55- 60- 65- 70- 75- 80- 85- > 90	62,877 62,713 45,058 19,635 17,218 15,516 13,337 12,481 8001 5639 4426 3529	70 83 81 80 138 120 123 120 90 65 40 38	11.1 13.2 18.0 40.7 80.2 77.3 92.2 96.2 112.5 115.3 90.4 107.7
Men	20- 30- 40- 50 55- 60- 65- 70- 75- 80- 85- > 90	56,787 66,623 50,044 21,138 17,998 15,071 12,408 11,529 6587 3757 2340 1268	56 44 66 26 28 38 26 27 20 11 2 3	9.9 6.6 13.2 12.3 15.6 25.2 21.0 23.4 30.4 29.3 8.6 23.7

**Fig. 1:**
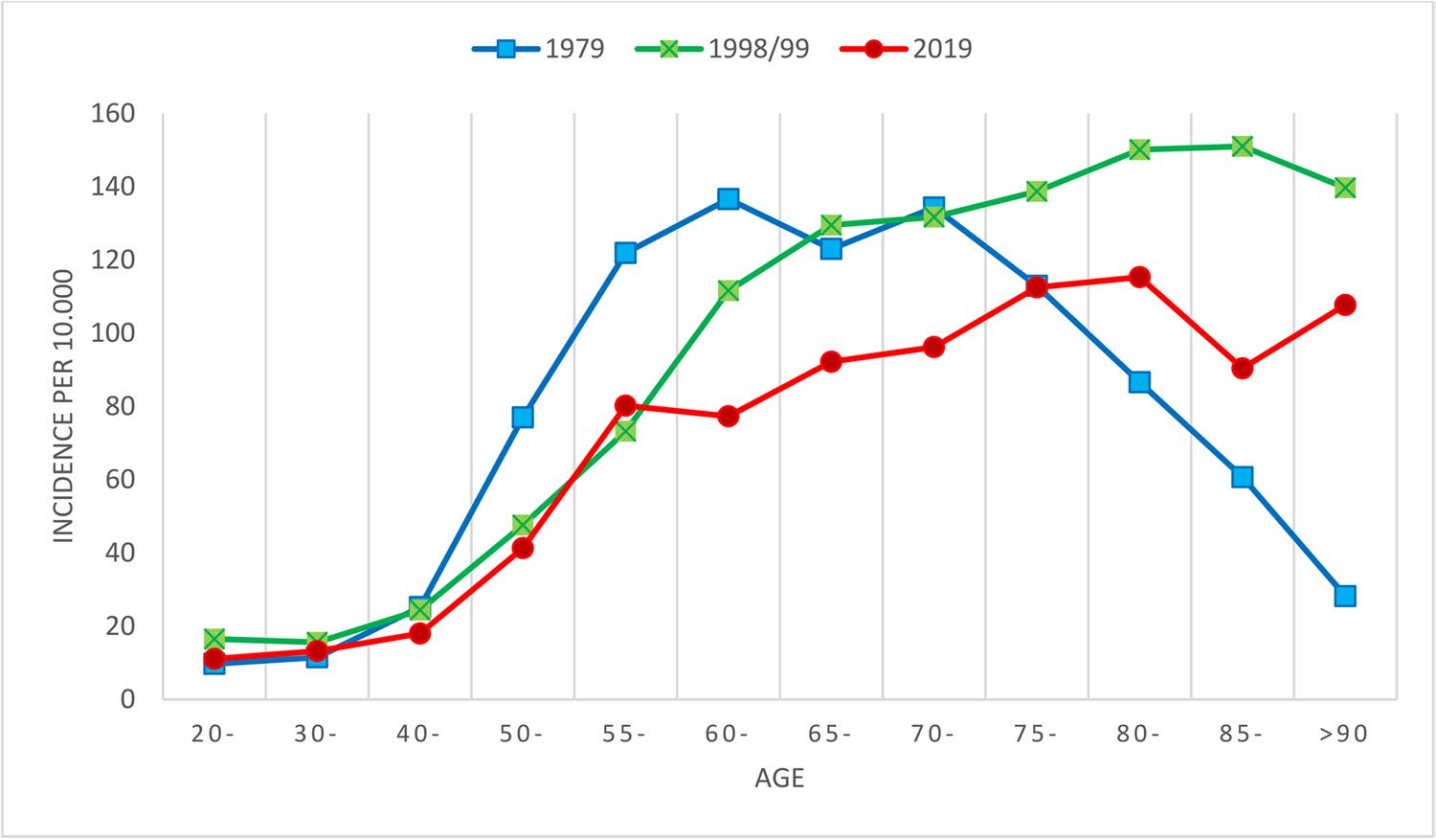
The incidence rate of distal radius fractures in 2019 in different age groups in women compared to 1998/1999 and 1979

**Fig. 2:**
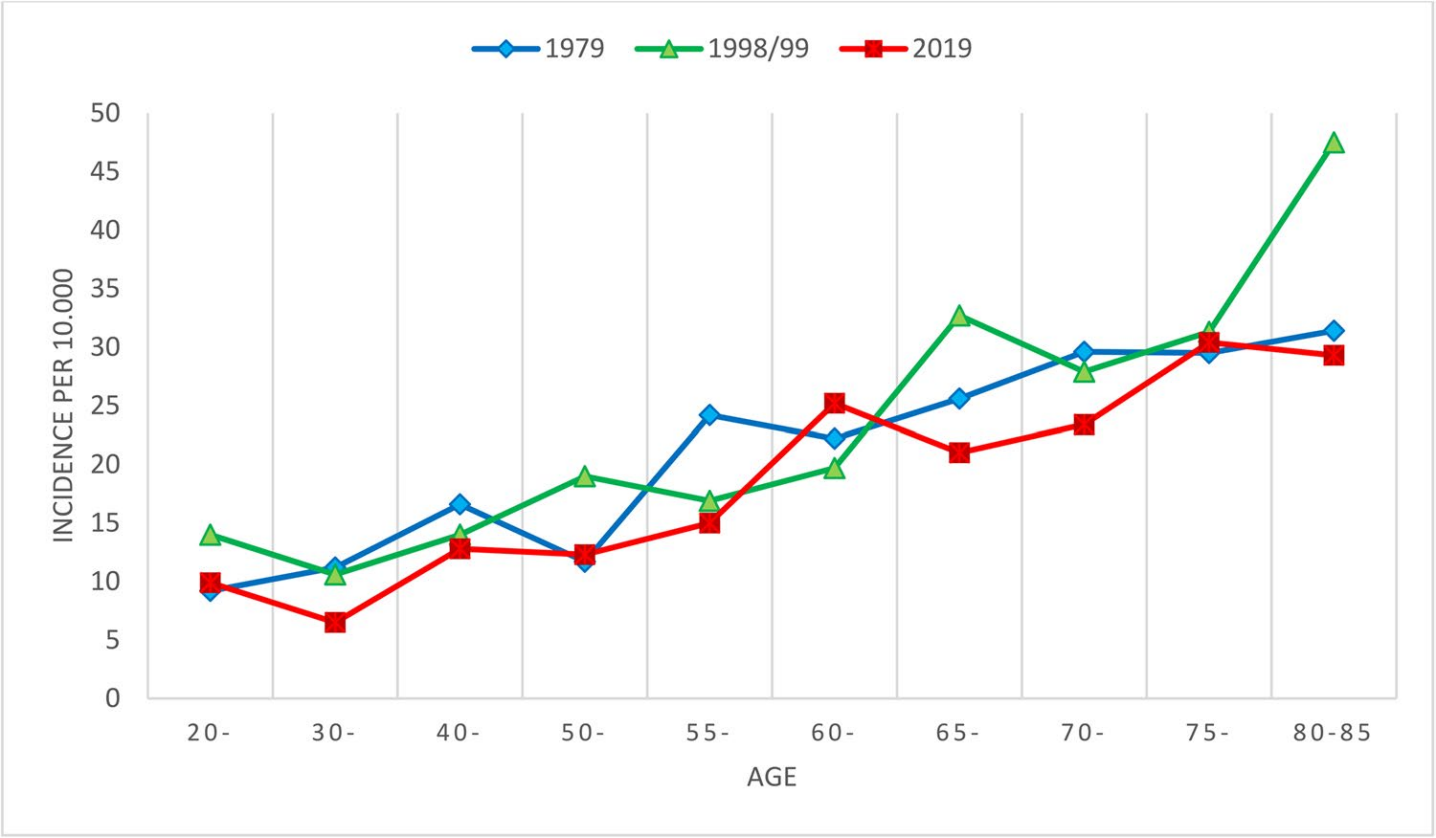
The incidence rate of distal radius fractures in 2019 in different age groups in men compared to 1998/1999 and 1979. The number of fractures in men > 85 was only 5, so the data are not presented

In women, the incidence increased sharply from age 50 to 54 with a more moderate increase from age 60 to 64. In 1998/1999 [11], the exponential increase continued up to the 65 years group (Fig. 1). In men, the incidence increased almost linear by age, but except for the youngest, the incidence in each age group was lower compared to women (Fig. 2).

The age-adjusted incidence rates per 10,000 observation years for women ≥ 50 years were 101.9 (95% CI 95.0–108.8) in 1998/1999 compared to 80.4 (95% CI 74.9–85.9) in 2019 with the corresponding figures in men being 24.6 (95% CI 20.7–28.4) in 1998/1999 and 19.9 (95% CI 17.0–22.8) in 2019.

The age-standardized IRR for women and men in the age group ≥ 50 years in 2019 was 0.78 (95% CI 0.71–0.86) and 0.78 (95% CI 0.63–0.97) compared to 1998/1999.

### Incidence in immigrants

The largest immigrant group from outside Europe is the people originating from Asia, representing 5% (*n* = 73) of the patients ≥ 20 years in 2019 compared to 3% (*n* = 40) in 1998/1999 (*p* = 0.001). Distal radius fractures in patients originating from Norway, i.e., born in Norway and both parents born in Norway represented 73% (*n* = 1020) in 2019 compared to 87% (*n* = 1302) in 1998/1999 (*p* < 0.001).

In both women and men, the age-adjusted incidence rates for the age group ≥ 50 years was lower in those originating from Asia compared to the majority population in Oslo (Table 2). In 2019, the age-standardized IRR of a distal radius fracture in the population in Oslo originating from Asia for the age group ≥ 50 years was 0.57 (95% CI 0.40–0.80) for women and 0.77 (95% CI 0.44–1.37) for men compared to the population in Oslo originating from Norway in the same age group. More than 99% of the patients ≥ 50 years with Asian origin included in our study were first-generation immigrants.

**Table 2:** The age-adjusted incidence rates (per 10,000 observation years) of distal radius fractures in the age-group ≥ 50 years

Geographical area	Year(s) of study	Female (95%CI)	Male (95%CI)	F/M ratio
Oslo, Norway	2019	80.4 (74.9–85.9)	19.9 (17.0–22.8)	4.0
Oslo, summer incidence	2019	61.4 (56.6–66.2)	14.4 (11.9–16.9)	4.3
Oslo, Asian origin	2019	40.9 (26.2–55.5)	15.3 (6.6–24.0)	2.7
Oslo, Norwegian origin	2019	84.3 (77.7–90.8)	21.4 (17.9–25.0)	3.9
Akershus, Norway	2011	52.7 (47.9–57.6)	13.1 (10.5–15.8)	4.0
Kristiansand, Norway	2004/2005	76.8 (71.1–82.5)	21.8 (18.5–25.1)	3.5
Skåne, Sweden	2016	52.3 (45.1–59.5)	12.0 (8.5–15.6)	4.4
Oulu, Finland	2008	70.0 (59.2–80.7)	23.7 (16.3–31.1)	3.0
Danmark	2010	89.3 (87.5–91.1)	20.7 (19.8–21.7)	4.3

The age-adjusted incidence rates vary between the different groups of immigrants for the age group > 50 years in Oslo (Table 3).

**Table 3:** The age-adjusted incidence rate (per 10,000 observation years) in different immigrant groups ≥ 50 years in Oslo

Geographical region of origin	Sex	Number of patients > 50 years	Population > 50 years in 2019	Age-adjusted incidence	95% confidence interval
Total	Female	814	99,782	80.4	74.9–85.9
	Male	181	92,096	19.9	17.0–22.8
Norwegian	Female	659	74,283	84.3	77.7–90.8
	Male	140	63,705	21.4	17.9–25.0
EU/EEA Countries	Female	83	5846	143.8	112.9–174.7
	Male	22	6602	34.1	18.8–49.3
Non-EU/EEA European countries	Female	10	2283	40.2	15.2–65.3
	Male	1	2194	4.4	0–13.1
Asia including Turkey	Female	35	8608	40.9	26.2–55.5
	Male	13	9491	15.3	6.6–24.0
Africa	Female	9	2457	32.8	7.0–58.6
	Male	3	3975	9.0	0–21.2
North America	Female	12	574	195.6	84.2–307.0
	Male	1	41	16.9	0–50.0
South and Central America	Female	7	926	76.5	15.6–137.4
	Male	0	867	0	0
Oceania	Female	0	41	0	0
	Male	0	58	0	0

## Discussion

We found a decrease in the number of distal radius fractures in 2019 compared to 1998/1999. In both women and men, the age-standardized IRR was decreased by more than 20% in 2019 compared to 1998/1999 for all adults and for those over 50 years of age.

### Comparison of incidence by region and country

For patients ≥ 50 years, we have compared the age-adjusted incidence rates with other Nordic countries and other Norwegian regions (Table 2).

Studies from Sweden [13] and Finland [14] showed a lower incidence in women. A study from Denmark back in 2010 showed a higher incidence in women than in Oslo in 2019 [15]. The incidence rate in Oslo’s neighboring county Akershus in a study from 2011 [16] was lower than in the present study. A study from the Southern part of Norway in 2004/2005 showed a slightly lower incidence in women, but not in men compared to Oslo in 2019 [18]. The incidence of distal radius fractures is higher in urban than in rural areas [19], and demographic differences between Oslo and other regions in Norway could have an impact here, since Oslo is a large city, and both Akershus and the Southern part of Norway consist of small cities, suburbs, and farmland. More women are living alone in Oslo than other places in Norway [17]. Living alone is linked to a higher incidence of fractures [20]. Slippery streets and less daylight during the winter contribute too many of the falls causing fracture [21]. Summer incidence could make a better comparison to other countries without the impact of snow and ice. The age-adjusted summer incidence for women and men in Oslo was found to be lower than the incidence during a whole year. The other Nordic countries also have winters with snow and ice though, so the high total incidence cannot be explained by these conditions only.

### How can we explain the decreasing incidence?

There may be several reasons for the decrease in incidence. A contribution may be that the climate has been warmer and the winters have been shorter during the last 30 years [22].

A study looking at causes for the declining hip fracture incidence in Norway between 1999 and 2019 found that increased physical activity, reduced smoking, reduced use of benzodiazepine, and increased osteoporotic treatment were all parts of the explanation [23]. Most of these factors may also have contributed to the reduced incidence in distal radius fractures.

During the last 20 years, there has been an increased focus on osteoporosis and prevention on falls in the older age groups. Fracture Liaison Service, a nurse-driven program, where patients above 50 years of age with a fracture are offered screening for osteoporosis, was implemented at Oslo University Hospital in 2015 [24]. However, the distal radius fracture is often the first osteoporotic fracture in patients above 50 years of age, especially in women, so secondary fracture prevention may not have the same effect compared to other type of fractures since treatment seldom is given before the patient has a fracture [2].

In 2015, a program of preventing falls in the elderly was started in some of the districts of Oslo and has since expanded to almost all the districts [25]. Falls in the elderly are an important risk factor for fractures [26], and thus preventing falls could have an impact on the decreased incidence.

The elderly population is more active [17] and healthier than in earlier years, which could improve both bone mass and neuromuscular control. This could work both ways. Active elderly people with more neuromuscular control and faster walking speed have a higher risk of distal radius fracture than more fragile elderly who lack the reaction time to stretching out the hand to stop a fall or loss of balance. Fragile elderlies are more likely to land on their hip or shoulder [27, 28].

Some studies have shown that the incidence of other types of osteoporotic fractures such as ankle, hip, proximal humerus, and knee fractures in the elderly increased until the mid-90 s and then stabilized [29–32]. The prevention of falls and osteoporosis had not been widely implemented at that time, so there are other and largely unknown factors for this stabilization of the incidence.

### Influence from immigration

The proportion of immigrants in Oslo from countries outside Europe and North America has increased from about 1% in 1979 to 13% in 1999 and up to 21% of the population in 2019 [17]. The largest group of these immigrants comes from Asia. In our study, people with Asian background ≥ 50 years had a lower incidence than people with Norwegian background. Thus, the increased number of people with different geographic region of origin in Oslo can contribute to the lower incidence in 2019 compared to 1998/1999. However, the incidence of the part of the population with Norwegian background was similar to the incidence of the total population in Oslo.

A recent study from Sweden showed that the rates of distal radius fractures of patients ≥ 20 years were marginally lower in immigrants of both first- and second-generation than native Swedes [33]. The rates of osteoporotic fractures in patients ≥ 50 years, however, were lower among first-generation immigrants compared to Swedish natives, while the rates in second-generation immigrants were similar [34, 35]. This could indicate that environmental factors contribute more to fragility fracture rates than ethnicity.

### Strengths and limitations

This study has some limitations: We cannot be sure if or how many patients could potentially have received treatment from hospitals outside Oslo. However, we believe that most of these patients would have been registered during follow-up, since almost all patients with a distal radius fracture would need one or more follow-up appointments for new radiology and/or remove the cast. Another limitation is that we did not examine all the radiographs at Volvat Medical Centre, Diakonhjemmet, and Akershus University Hospital. However, supposing that an equal proportion of fractures were misclassified as at Oslo University Hospital, this would give us an increase in the total number of fractures by 13 from 1395 to 1408, which is around 1% of the total number. However, by including other diagnosis codes than S52.5 and S52.6, we believe we have reduced this problem.

A strength of this study is that we scrutinized the medical records to verify the fractures as well as all relevant radiographs and CT scans at Oslo University Hospital who treated 90% of the patients in our study. This means that very few fractures were lost due to wrong diagnoses. Many studies reporting incidence rates use electronic diagnosis registers as their only data source. This may lead to a lower reliability because it relies on the physicians to report the correct diagnosis. In our study, 9% of the patients with a distal radius fracture treated at Oslo University Hospital did not have the correct electronic diagnosis code (S52.5, S52.6). Using radiographic examinations and medical records in addition to electronic diagnosis codes to verify the diagnosis makes the incidence rates more reliable. In addition, we used the same method as the previous studies from Oslo [10, 11], making the comparison over the decades more accurate. This implies that our findings are real, and not an expression of lost data.

## Conclusion

The incidence of distal radius fractures has decreased in Oslo over the last 20 years in all adults and more pronounced in those ≥ 50 years. The incidence in Oslo is still higher than in other regions of Norway and in some of the other Nordic countries.

## Data Availability

Due to protection of privacy under General Data Protection Regulation and Norwegian law, the individual-level data can only be made available after approval by the Regional Committee for Medical and Health Research Ethics.
